# Increasing glycaemia is associated with a significant decline in HDL cholesterol in women with prediabetes in two national populations

**DOI:** 10.1038/s41598-021-91075-9

**Published:** 2021-06-09

**Authors:** Chaiwat Washirasaksiri, Weerachai Srivanichakorn, Ian F. Godsland, Chayanis Kositamongkol, Suwat Chariyalertsak, Pattapong Kessomboon, Sawitri Assanangkornchai, Surasak Taneepanichskul, Nareemarn Neelapaichit, Pochamana Phisalprapa, Desmond G. Johnston, Nick S. Oliver, Wichai Aekplakorn

**Affiliations:** 1 Department of Medicine, Faculty of Medicine Siriraj Hospital, Mahidol University, 2 Wang Lang Road, Bangkok Noi, Bangkok, 10700 Thailand; 2 Department of Metabolism, Digestion and Reproduction, Faculty of Medicine, Imperial College, London, SW7 2AZ UK; 3 Faculty of Public Health, Chiang Mai University, Chiang Mai, Thailand; 4 Faculty of Medicine, Khon Kaen University, Khon Kaen, Thailand; 5 Epidemiology Unit, Faculty of Medicine, Prince of Songkla University, Songkhla, Thailand; 6 College of Public Health Sciences, Chulalongkorn University, Bangkok, Thailand; 7 Ramathibodi School of Nursing, Faculty of Medicine, Ramathibodi Hospital, Mahidol University, Bangkok, Thailand; 8 Department of Community Medicine, Faculty of Medicine Ramathibodi Hospital, Mahidol University, Rama VI Rd., Ratchathewi, Bangkok, Thailand

**Keywords:** Endocrinology, Risk factors

## Abstract

Internationally, studies have shown associations between lipids and glycemia; however, whether the link varies by gender and population has been rarely examined. We investigated relationships between glycemia and HDL- and Non-HDL-cholesterol and their modification by gender. We undertook a cross-sectional analysis from the National Health Examination Survey for Thailand (NHES-Thailand) and the Health Survey for England (HS-England) in adults aged 18–75 year. Glycaemia was assessed by FPG in Thailand and by HbA1c in the UK. In population- and gender-stratified analyses, the relationships between glycemia and lipids were explored. A total of 15,145 Thai and 3484 UK adults with blood measurement were included. The prevalences of prediabetes were: in NHES-Thailand, 16% (SE = 0.004), based on FPG (5.6 to < 7.0 mmol/L) and in HS-England, 19% (0.007) based on HbA1c (39 to < 48 mmol/mol). Increasingly abnormal glucose homeostasis was associated with increasing age, adiposity, SBP, proportion of antihypertensive and lipid-lowering agent use and with decreasing HDL-cholesterol. Independent of age, adiposity, smoking, alcohol, physical activity, and lipid and BP lowering drug use, increasing glycemia was associated with decreasing HDL-cholesterol specifically in women with prediabetes (NHES-Thailand, beta-coefficient − 0.07 (95% CI − 0.15, − 0.001) p = 0.04 and HS-England, − 0.03 (− 0.04, − 0.006) p = 0.01). In both populations, among those with prediabetes, increasing glycaemia is associated with an adverse, significant decline in HDL cholesterol, specifically in women. These adverse effects are apparent in widely-differing international populations.

## Introduction

Cardiovascular disease (CVD) is presently the most important cause of mortality and disability in people with or without diabetes worldwide^1–3^. Hyperglycemia and dyslipidemia are among the major contributors to CVD risk^4,5^. Among features of dyslipidemia, low HDL cholesterol and high non-HDL cholesterol are a pattern common not only in diabetes^6^ but also in lesser disturbances in glucose homeostasis, commonly referred to as ‘prediabetes’^7–9^. Abnormal glucose homeostasis status may be characterized by HbA1c, fasting plasma glucose (FPG) or oral glucose tolerance test 2-h plasma glucose concentrations (OGTT-2 h glucose) elevated above defined thresholds. In contrast to OGTT-2 h glucose, HbA1c and FPG have several advantages for establishing prediabetes^10–12^. Consequently, FPG and HbA1c are commonly implemented in real world studies.

Although previous meta-analysis and studies have found low agreement between individual glycaemia classifications derived from FPG, HbA1c and OGTT-2 h glucose, the overall prevalence estimates for prediabetes based on ADA criteria for FPG or HbA1c are similar, but this is not the case for OGTT-2 h glucose. Accordingly, in a meta-analysis of glycaemia screening reports, the overall prevalences for prediabetes were 61% according to FPG 5.6–6.9 mmol/L, 68% according to HbA1c 39–47 mmol/mol but only 15.6% according to OGTT-2 h glucose 7.8–11.1 mmol/L^13^. Studies in at-risk, multiethnic populations using FPG criteria as reference standard for prediabetes found that HbA1c had positive predictive values of between 0.55 and 0.75^14–16^. Importantly, meta-analysis of population-based, prospective cohort studies has shown the association of prediabetes with cardiovascular disease risk, whether prediabetes was defined by OGTT impaired glucose tolerance, impaired fasting glucose, or raised HbA1c. Therefore, although FPG and HbA1c may identify different individuals as having prediabetes, at the population level, there is marked overlap and the two measures can provide similar information about glycaemia and disease risk.

A number of studies have demonstrated that, over the full range of glycemia, increasing FPG is associated with an increasingly atherogenic lipid and lipoprotein profile^17,18^. However, there is little information on how consistent this relationship is internationally, particularly for countries with widely different ethnicity and climatic and cultural characteristics. In the absence of in-depth comparisons of methodology, between-country comparisons of absolute risk factor levels can be compromised by lack of common standardization. Nevertheless, differences in standardization need not disrupt the comparability between within-survey measures of risk factor inter-relationship. Accordingly, in the present study, we have drawn on population survey data from the National Health Examination Survey for Thailand (NHES-Thailand) 2014 and the Health Survey for England (HS-England) 2014 and investigated relationships between glycemia and high density lipoprotein (HDL-), and non-HDL cholesterol and their modification by gender.

## Results

For the NHES-Thailand 2014, 22,217 participants and for the HS-England 2014, 10,080 participants were interviewed. Of those, 15,145 and 3,484 participants, respectively, aged 18–75 years and with age, gender, glycemia and lipid measurements were included in the present analysis. Overall, participants (male 46% (NHES-Thailand) and 42% (HS-England)) had a mean age of 51.2 (0.2) and 50.9 (0.2) years in NHES-Thailand and of 49.1 (0.4) and 49.2 (0.3) years in HS-England in males and females, respectively. The prevalence of IGH was 16% (0.004) in NHES-Thailand and were 19% (0.007) in HS-England (Table 1).

**Table 1:** Fasting Plasma Glucose and HbA1c level by glycemic categories in two study populations.

Parameters		NGH	IGH—mild	IGH—severe	Diabetes
**NHES-Thailand (% (SEP))**		FPG < 5.6	FPG 5.6 to < 6.1	FPG 6.1 to < 7.0	Diabetes*
		74 (0.01)	11 (0.004)	5 (0.003)	10 (0.003)
Gender** (% (SEP))	M	48 (0.01)	50 (0.02)	52 (0.03)	43 (0.02)
	F	52 (0.01)	50 (0.02)	48 (0.03)	57 (0.02)
FPG, mmol/L (% ± SEM)	M	5.0 ± 0.02	5.8 ± 0.01	6.4 ± 0.02	9.0 ± 0.21
	F	4.9 ± 0.01	5.8 ± 0.01	6.4 ± 0.02	9.1 ± 0.14
**HS-England (% (SEP))**		HbA1c < 39	HbA1c 39 to < 42	HbA1c 42 to < 48	Diabetes*
		75 (0.01)	13 (0.01)	6 (0.004)	6 (0.004)
Gender*** (% (SEP))	M	49 (0.01)	49 (0.02)	48 (0.03)	61 (0.04)
	F	51 (0.01)	51 (0.02)	52 (0.03)	39 (0.04)
HbA1c, (% ± SEM) (mmol/mol)	M	5.3 ± 0.01%	5.8 ± 0.01%	6.1 ± 0.02%	7.6 ± 0.2%
		34.0 ± 0.1	39.8 ± 0.1	43.3 ± 0.2	59.9 ± 1.7
	F	5.3 ± 0.01%	5.8 ± 0.01%	6.2 ± 0.01%	8.1 ± 0.2%
		34.1 ± 0.1	39.8 ± 0.1	43.5 ± 0.1	65.0 ± 2.2

FPG and HbA1c are presented as mean ± standard error of mean, SEM (%) and gender proportion as % (standard error of proportion, SEP).

*NGH* normal glucose homeostasis, *IGH* impaired glucose homeostasis, *NHES-Thailand* the National Health Examination Survey for Thailand, *HS-England* the Health Survey for England, *HbA1c* hemoglobin A1c, *FPG* fasting plasma glucose, *n* the number, *M* male, *F* female, *p* p value.

*Diabetes is defined by Participants with FPG ≥ 7.0 mmol/L (Thai) and/or HbA1c ≥ 48 mmol/mol (UK) and/or current on glucose lowering agent and/or self-reported previous diagnosis by doctor.

**Significant variation between gender proportion p = 0.01.

***Significant variation between gender proportion p = 0.02.

In both NHES-Thailand and HS-England, increasing glycemia was associated with increasing age, BMI, and waist circumference and with higher proportions of BP and lipid lowering agent use (Table 2). Although some significant variation was observed, trends in non-HDL cholesterol between glycaemia categories were inconsistent. However, HDL cholesterol fell consistently with increasing degrees of abnormal glucose homeostasis in both NHES-Thailand and HS-England men and women. Interestingly, the means of age-standardized HDL cholesterol in UK English with NGH were higher than those in Thais (15.4% and 23.3% higher, in males and females, respectively) and accordingly, the proportions of low HDL-cholesterol in Thai were 3–4 times higher than those in UK English participants. However, the reduction in HDL cholesterol with increasing glycaemia was greater in UK English than that in Thais, especially in the IGH-severe (age-standardized HDL cholesterol reduction, − 10.2% vs − 5.2% (male) and − 14.6% vs − 8.8% (female) and in diabetes (− 19.9% vs − 10.6% (male) and − 13.6% vs − 5.6% (female). Consequently, although the proportions of age-standardized low HDL- cholesterol in Thai were higher than in UK English participants in both genders, these differences decreased with increasing glycemia (Supplementary Table S1).

**Table 2:** Crude means and proportion of individual characteristics and lipid level by glycemic categories in two study populations.

Parameters	NHES-THAILAND					HS-ENGLAND				
	NGH	IGH—mild	IGH—severe	Diabetes	p*	NGH	IGH—mild	IGH—severe	Diabetes	p*
**Age, years**										
Male	41.5 ± 0.3	46.8 ± 0.7	50.3 ± 0.9	52.7 ± 0.7	< 0.001	40.6 ± 0.5	53.7 ± 1.0	56.2 ± 1.2	56.6 ± 1.2	< 0.001
Female	42.2 ± 0.3	48.3 ± 0.7	49.7 ± 1.1	53.5 ± 0.6	< 0.001	41.1 ± 0.5	54.4 ± 0.9	59.2 ± 1.1	56.4 ± 1.5	< 0.001
**BMI, kg/m**^**2**^										
Male	23.5 ± 0.1	24.6 ± 0.3	25.0 ± 0.3	25.0 ± 0.2	< 0.001	26.5 ± 0.2	28.6 ± 0.3	29.2 ± 0.4	30.6 ± 0.7	< 0.001
Female	24.5 ± 0.1	25.8 ± 0.2	26.6 ± 0.4	26.4 ± 0.2	< 0.001	26.3 ± 0.2	28.7 ± 0.4	29.4 ± 0.6	33.1 ± 0.8	< 0.001
**Waist circumference, cm**										
Male	82.2 ± 0.3	85.6 ± 0.7	86.8 ± 0.9	87.7 ± 0.6	< 0.001	93.8 ± 0.5	101.4 ± 0.9	104.0 ± 1.2	106.1 ± 1.6	< 0.001
Female	80.4 ± 0.2	84.4 ± 0.6	86.0 ± 1.0	87.7 ± 0.5	< 0.001	84.7 ± 0.4	92.5 ± 0.9	95.7 ± 1.3	102.2 ± 1.6	< 0.001
**Antihypertensive drug use (%)**										
Male	7 (0.01)	11 (0.01)	20 (0.03)	31 (0.02)	< 0.001	7 (0.01)	23 (0.03)	22 (0.04)	52 (0.05)	< 0.001
Female	7 (0.01)	11 (0.01)	20 (0.03)	31 (0.02)	< 0.001	6 (0.01)	18 (0.02)	32 (0.04)	52 (0.06)	< 0.001
**Lipid lowering agent drug use (%)**										
Male	4 (0.004)	6 (0.01)	10 (0.02)	26 (0.02)	< 0.001	5 (0.01)	20 (0.03)	34 (0.05)	68 (0.05)	< 0.001
Female	6 (0.004)	12 (0.01)	16 (0.02)	35 (0.02)	< 0.001	4 (0.01)	13 (0.02)	24 (0.04)	58 (0.06)	< 0.001
**Current smoking (%)**										
Male	5 (0.01)	20 (0.03)	34 (0.05)	68 (0.05)	< 0.001	5 (0.01)	20 (0.03)	34 (0.05)	68 (0.05)	< 0.001
Female	5 (0.01)	20 (0.03)	34 (0.05)	68 (0.05)	< 0.001	5 (0.01)	20 (0.03)	34 (0.05)	68 (0.05)	< 0.001
**Current alcoholic drinking (%)**										
Male	59 (0.01)	67 (0.02)	59 (0.03)	50 (0.03)	< 0.001	89 (0.01)	86 (0.03)	84 (0.04)	75 (0.05)	< 0.001
Female	26 (0.01)	23 (0.02)	22 (0.03)	14 (0.02)	< 0.001	84 (0.01)	77 (0.03)	78 (0.04)	64 (0.06)	< 0.001
**Physically active (%)**										
Male	82 (0.01)	81 (0.02)	84 (0.02)	79 (0.02)	0.3	68 (0.02)	54 (0.04)	51 (0.05)	37 (0.05)	< 0.001
Female	82 (0.01)	85 (0.02)	84 (0.02)	81 (0.02)	0.1	53 (0.02)	46 (0.03)	39 (0.04)	27 (0.05)	< 0.001
**Non-HDL Chol, mmol/L**										
Male	3.85 ± 0.03	3.98 ± 0.07	4.10 ± 0.08	3.91 ± 0.08	0.04	3.69 ± 0.04	4.02 ± 0.08	3.97 ± 0.12	3.20 ± 0.12	0.2
Female	3.83 ± 0.02	4.12 ± 0.06	4.02 ± 0.08	3.95 ± 0.06	0.001	3.33 ± 0.03	4.05 ± 0.07	3.90 ± 0.10	3.56 ± 0.15	< 0.001
**HDL cholesterol, mmol/L**										
Male	1.23 ± 0.01	1.14 ± 0.02	1.16 ± 0.02	1.10 ± 0.02	< 0.001	1.42 ± 0.01	1.34 ± 0.03	1.30 ± 0.04	1.18 ± 0.04	< 0.001
Female	1.36 ± 0.01	1.31 ± 0.02	1.28 ± 0.03	1.25 ± 0.02	< 0.001	1.69 ± 0.01	1.65 ± 0.03	1.55 ± 0.04	1.34 ± 0.05	< 0.001

*HS-England* the Health Survey for England, *p* p value, *BMI* body mass index, *HDL-c* High density lipoprotein cholesterol, *LDL-c* Low density lipoprotein cholesterol.

*Summary statistics for raw data are presented, unstandardized for any between-population differences. Continuous normally-distributed variables; mean ± standard error of mean, Categorical variables; percentage (standard error of the proportion) are shown. NGH; normal glucose homeostasis (FPG < 5.6 mmol/L (Thai) or HbA1c < 5.7% (UK)), IGH-mild; mild impaired glucose homeostasis (FPG 5.6 to < 6.1 mmol/L (Thai) or HbA1c 39 to < 42 mmol/mol (UK)), IGH-severe: severe impaired glucose homeostasis (FPG 6.1 to < 7.0 mmol/L (Thai) or HbA1c 42 to < 48 mmol/mol (UK)), diabetes, FPG ≥ 7.0 mmol/L or HbA1c ≥ 48 mmol/mol and/or currently taking a glucose lowering agent and/or self-reported previous diabetes diagnosis by doctor, the National Health Examination Survey for Thailand.

**Figure 1:**
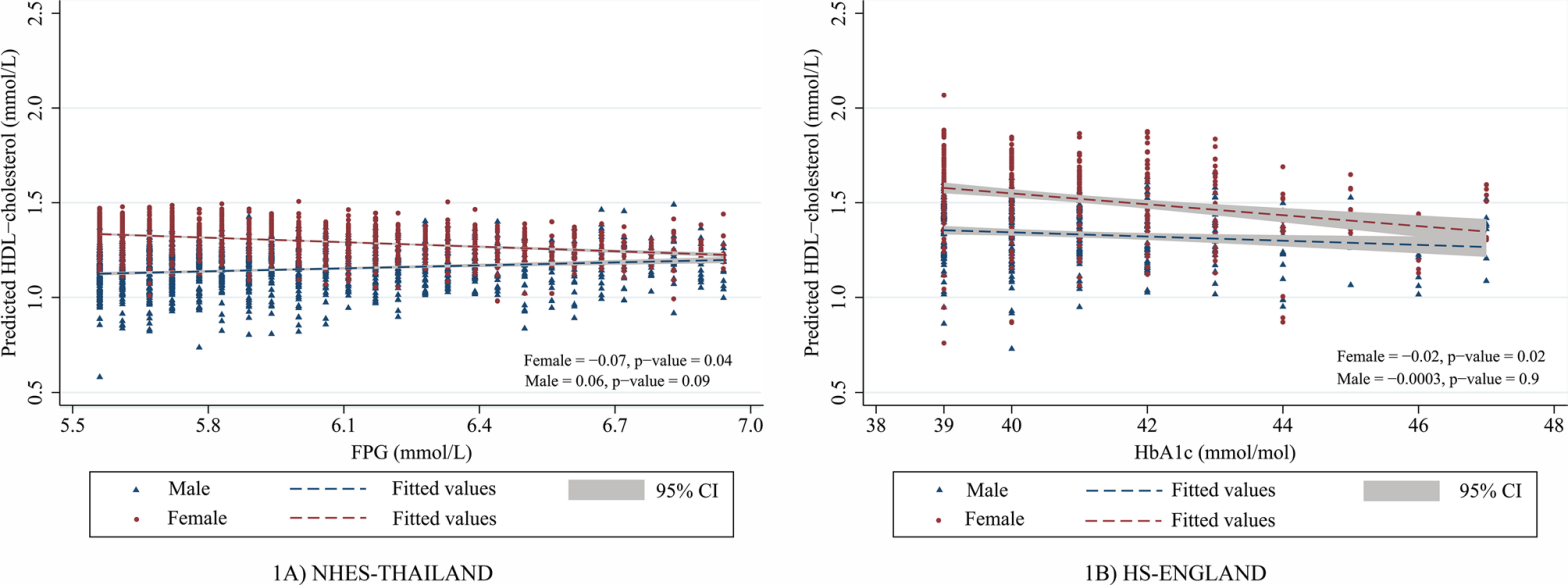
Regression lines fitted to the model-predicted HDL-c concentrations from the models reported in Tables 3 and 4 to illustrate the differences in strengths of association between HDL-c and glycaemia between men and women with impaired glucose homeostasis in the National Health Examination Survey for Thailand ((**A**), NHES-Thailand) and in the Health Survey for England ((**B**), HS-England).

In gender separated analysis (Table 3 for NHES-Thailand, Table 4 for HS-England, Fig. 1), glycemia as an independent predictor of HDL-c and non-HDL-c was explored with inclusion of the individual characteristics: age, waist circumference, lipid lowering agent use, current smoking, alcoholic drinking, and physical activity status as predictor variables in multivariable linear regression models. Overall, with all glycaemia categories included, but specifically in women, HDL cholesterol decreased independently with either increasing FPG in Thais (coefficient (95% CI) − 0.011 (− 0.017, − 0.004) mmol/L, p = 0.001) or HbA1c in UK English (− 0.003 (− 0.006, − 0.0004), p = 0.02), and this association was specifically apparent in the impaired glucose homeostasis range (NHES-Thailand: − 0.073 (− 0.150, − 0.0002), p = 0.04, and HS-England: − 0.021 (− 0.04, − 0.003), p = 0.02). Overall, with all glycaemia categories included, in both men and women and in both Thailand and the UK, non-HDL cholesterol was independently associated with increasing glycaemia. This association was also apparent in diabetes and NGH (only female) in Thai study, and in NGH in UK study.

**Table 3:** Regression coefficients (95% CI) of FPG and covariates associated with HDL-c and Non HDL-c by glycemic categories and gender in the Thai National Health Examination Survey**.**

NHES-Thailand	Male				Female			
*HDL-c*	Overall	NGH	IGH	Diabetes	overall	NGH	IGH	Diabetes
FPG	− 0.005 (− 0.014, 0.005)^0.3^	0.002 (− 0.017, 0.022)^0.8^	0.063 (− 0.011, 0.138)^0.09^	0.011 (− 0.002, 0.023)^0.08^	− 0.011 (− 0.017, − 0.004)^0.001^	− 0.025 (− 0.045, − 0.005)^0.01^	− 0.073 (− 0.150, − 0.0002)^0.04^	− 0.002 (− 0.010, 0.006)^0.6^
Age	− 0.001 (− 0.002, 0.00001)^0.05^	− 0.001 (− 0.002, 0.001)^0.05^	0.001 (− 0.001, 0.003)^0.1^	0.001 (− 0.002, 0.005)^0.5^	0.00004 (− 0.001, 0.001)^0.9^	− 0.0001 (− 0.001, 0.001)^0.8^	0.002 (− 0.0001, 0.004)^0.06^	− 0.0006 (− 0.004, 0.002)^0.7^
Waist circumference	− 0.008 (− 0.009, − 0.007^)<0.001^	− 0.008 (− 0.010, − 0.007)^<0.001^	− 0.008 (− 0.011, − 0.006)^<0.001^	− 0.004 (− 0.007, − 0.001)^0.04^	− 0.007 (− 0.008, − 0.006)^<0.001^	− 0.008 (− 0.009, − 0.007)^<0.001^	− 0.005 (− 0.007, − 0.003)^<0.001^	− 0.006 (− 0.009, − 0.003)^<0.001^

*Non HDL-c*	Overall	NGR	IGH	Diabetes	Overall	NGR	IGH	Diabetes
FPG	0.034 (0.004, 0.065)^0.02^	0.057 (− 0.024, 0.139)^0.1^	0.072 (− 0.178, 0.323)^0.5^	0.078 (0.030, 0.127)^0.02^	0.050 (0.028, 0.072)^<0.001^	0.161 (0.100, 0.222)^<0.001^	− 0.099 (− 0.309, 0.111)^0.3^	0.088 (0.058, 0.118)^<0.001^
Age	0.007 (0.004, 0.010)^<0.001^	0.010 (0.006, 0.013)^<0.001^	0.002 (− 0.005, 0.010)^0.5^	− 0.010 (− 0.022, 0.003)^0.1^	0.014 (0.012, 0.017)^<0.001^	0.014 (0.011, 0.017)^<0.001^	0.014 (0.0007, 0.021)^<0.001^	0.011 (0.002, 0.020)^0.01^
Waist circumference	0.025 (0.021,0.030)^<0.001^	0.024 (0.020, 0.029)^<0.001^	0.031 (0.021, 0.041)^<0.001^	0.023 (0.011, 0.036)^<0.001^	0.014 (0.011, 0.017)^<0.001^	0.014 (0.010, 0.017)^<0.001^	0.012 (0.005, 0.019)^0.001^	0.016 (0.007, 0.026)^<0.001^

*FPG* fasting plasma glucose, *BMI* body mass index, *HDL-c* high density lipoprotein cholesterol, *NGH* normal glucose homeostasis (FPG < 5.6 mmol/L), *IGH* impaired glucose homeostasis (FPG 5.6 to < 7.0 mmol/L), diabetes, FPG ≥ 7.0 mmol/L and/or currently taking a glucose lowering agent and/or self-reported previous diabetes diagnosis by doctor. In addition to those shown, predictor variables included in each multivariable model also included lipid lowering agent use, smoking, alcohol status, total energy intake and physical activity status.

**Table 4:** Regression coefficients (95% CI) of HbA1c and covariates associated with HDL-c and Non HDL-c by glycemic categories and gender in the Health Survey for England**.**

HS-England	Male				Female			
*HDL-c*	Overall	NGH	IGH	Diabetes	Overall	NGH	IGH	Diabetes
HbA1c	− 0.003 (− 0.006, − 0.001)^0.008^	0.005 (− 0.004, 0.013)^0.2^	− 0.0003 (− 0.021, 0.021)^0.9^	− 0.001 (− 0.005, 0.003)^0.5^	− 0.003 (− 0.006, − 0.0004)^0.02^	− 0.006 (− 0.014, 0.003)^0.1^	− 0.021 (− 0.04, − 0.003)^0.02^	0.002 (− 0.002, 0.006)^0.2^
Age	0.004 (0.003, 0.006^)<0.001^	0.005 (0.003, 0.007)^<0.001^	0.002 (− 0.002, 0.007)^0.3^	− 0.001 (− 0.006, 0.004)^0.7^	0.009 (0.008, 0.010)^<0.001^	0.010 (0.009, 0.012)^<0.001^	0.008 (0.005, 0.011)^<0.001^	0.0002 (− 0.008, 0.008)^0.9^
Waist circumference	− 0.010 (− 0.012, − 0.008)^<0.001^	− 0.011 (− 0.013, − 0.008)^<0.001^	− 0.01 (− 0.013, − 0.006)^<0.001^	− 0.007 (− 0.012, − 0.002)^0.006^	− 0.014 (− 0.016, − 0.013)^<0,001^	− 0.014 (− 0.016, − 0.013)^<0.001^	− 0.013 (− 0.016, − 0.011)^<0.001^	− 0.006 (− 0.015, 0.003)^0.1^

*Non HDL-c*	Overall	NGR	IGH	Diabetes	Overall	NGR	IGH	Diabetes
HbA1c	0.004 (− 0.008, 0.015)^0.5^	0.033 (0.009,0.057)^0.008^	− 0.001 (− 0.059,0.056)^0.9^	0.016 (− 0.003, 0.034)^0.1^	0.010 (0.002, 0.02)^0.01^	0.050 (0.030, 0.070)^<0.001^	− 0.015 (− 0.061, 0.032)^0.5^	− 0.005 (− 0.018, 0.008)^0.4^
Age	0.011 (0.007, 0.015)^<0.001^	0.012 (0.006, 0.017)^<0.001^	− 0.007 (− 0.019,0.006)^0.2^	− 0.013 (− 0.031, 0.005)^0.1^	0.027 (0.024, 0.031)^<0.001^	0.024 (0.019, 0.028)^<0.001^	0.020 (0.010, 0.030)^<0.001^	0.006 (− 0.032,0.045)^0.7^
Waist circumference	0.030 (0.026, 0.038)^<0.001^	0.036 (0.030, 0.042)^<0.001^	0.022 (0.010,0.033)^<0.001^	0.015 (0.001, 0.030)^0.04^	0.018 (0.014, 0.022)^<0.001^	0.018 (0.013, 0.022)^<0.001^	0.017 (0.010, 0.020)^<0.001^	0.008 (− 0.012,0.028)^0.4^

*HbA1c* Hemoglobin A1c, *BMI* body mass index, *HDL-c* high density lipoprotein cholesterol, *NGH* normal glucose homeostasis (HbA1c < 5.7%), *IGH* impaired glucose homeostasis (HbA1c 39 to < 42 mmol/mol), diabetes; HbA1c ≥ 48 mmol/mol and/or currently taking a glucose lowering agent and/or self-reported previous diabetes diagnosis by doctor. In addition to those shown, predictor variables included in each multivariable model also included lipid lowering agent use, smoking, alcohol status, total energy intake and physical activity status.

In addition to the independent associations between HDL cholesterol and glycaemia, in Thai people with impaired glucose homeostasis, decreasing HDL cholesterol with increasing waist circumference in both gender (Table 3). There were also significant independent associations between increasing HDL cholesterol and alcohol drinking in both genders and decreasing HDL cholesterol with current smoking in men (results not shown). In UK English people with impaired glucose homeostasis, increasing HDL cholesterol was associated with increasing age in women and decreasing HDL cholesterol was independently associated with increasing waist circumference in both genders (Table 4). Increasing HDL cholesterol was also associated with alcohol drinking and active physical activity in men and decreasing HDL cholesterol was associated with current smoking in both genders (results not shown). After stratification by lipid lowering agent use, in both UK and Thai, the directionality of the relationships and the magnitude of the coefficients between HDL-c and glycemia were similar, although with smaller numbers the statistical significances observed in the data as a whole and in the non-LLA users were lost among the LLA users (Supplement Tables S2–S5).

## Discussion

Adverse associations between HDL cholesterol and deteriorating glycaemia have been reported in a number of studies but we believe the present study is among very few to demonstrate these associations in population surveys from countries differing markedly in ethnicity, climate and culture. Increasing glycemia in each range of deteriorating glucose homeostasis was associated with increasingly adverse cardiometabolic risk characteristics, particularly age and adiposity, and with higher proportions of people taking BP- and lipid lowering agents. However, in women, both in Thai and English populations, deterioration in HDL cholesterol concentrations with increasing glycaemia was independent of these characteristics and was most apparent in women with impaired glucose homeostasis. We also found in both genders that, although the age standardized mean HDL cholesterol in the UK English population was higher than that in the Thai population with NGH, the magnitude of the reduction in the age standardized mean HDL cholesterol in the severe IGH and diabetes categories was approximately twice as great in the UK English than in the Thai population.

An increasingly adverse lipid risk factor profile with increasing severity of abnormal glucose homeostasis accords with the preceding Thailand National Health Examination Survey IV (NHES IV)^19^ and previous European and Asian population studies of relationships between cardiometabolic risk and glycemia^20–23^. Regarding the gender-specific association we observed, it is noteworthy that in an analysis from the multiethnic population studies, DECODA & DECODE (Diabetes Epidemiology: Collaborative analysis of Diagnostic criteria in Asia and in Europe), in people without a prior history of diabetes, the inverse relationship between HDL cholesterol levels and glycemia was only apparent in European women^17^, whereas the positive relationship between glycemia and non-HDL cholesterol was apparent overall in European and in almost all Asian ethnic groups studied^17,18^. Similarly, with more intensive risk factor adjustment, we found that among the Thai and UK English populations across the full range of glycemia, there were the same relationships between glycemia and HDL- or non-HDL cholesterol. Our observations are also consistent with our previous research in a Thai clinical sample at high cardiometabolic risk and with prediabetes defined by FPG and/or HbA1c, which found HDL cholesterol tended to fall with glycemia in women but not in men after taking all relevant factors into account^24^. It also accords with previous clinical studies that have found the adverse effect of increasing glycemia on the CVD risk profiles to be greater in women than in men^25,26^.

Although our analysis was primarily focused on relationships between HDL cholesterol and glycaemia, it should be noted that we found a higher proportion of low HDL cholesterol in our Asian relative to our European data, with a 3–4 times higher proportion of low HDL cholesterol in Thai people than in that in UK English people for each glucose homeostasis group. This accords with data from the DECODA and DECODE studies, in which, relative to Europeans, adjusted odds ratios for having lower HDL cholesterol were significantly higher for Indians, Hong Kong Chinese population^27^, which accords with our observations. These findings call into question the application to Asian populations of cut offs for HDL cholesterol developed in Western populations. Clearly, the relationship between HDL cholesterol and cardiovascular risk should be investigated further in Thai and South-East Asian people to establish whether or not the same relationship obtains between HDL cholesterol and cardiovascular disease as does in Western populations^28^. Interestingly, the impact of glycemia on HDL cholesterol appears greater in UK than in Thailand, with greatest impact being in women. In a further parallel with our observations, the DECODE study of ethnicity in a diverse non-diabetes population sample found that at any given state of glucose homeostasis the absolute CVD risk was higher in men than in women, but this difference narrowed when progressing from normoglycemic to impaired glucose homeostasis and newly diagnosed diabetes^29^. In general, in impaired glucose homeostasis or on developing diabetes, cardiovascular disease risk in women does appear to converge with that in men, and the relative protection from cardiovascular disease seen in normoglycemic women compared with normoglycemic men is lost^30–32^. Several mechanisms underlying this loss of protection have been suggested, including the possibility that diabetes may take longer to become apparent in women, resulting in longer exposure to the risk factor disturbances associated with impaired glucose homeostasis^33^. However, a definitive cause for the gender difference remains to be established.

Our analysis has strengths and limitations. To the best of our knowledge, this is the first study to explore the extent to which variation in glycemic parameters is related to lipids trans-nationally in Thai and UK English populations, independently of a range of potential confounders, namely age, waist circumference, lipid lowering agent use, current smoking, drinking and active physical status. Our study's principal limitation was that different glycemic parameters were measured in the two population samples: FPG in NHES-Thailand and HbA1c in HS-England. However, there is evidence for good comparability between FPG and HbA1c^14–16^ and similar prevalence of IGH and relationships between IGH and cardiovascular risk factors have been reported using either measure. Importantly, methodological differences in classifying degrees of glycaemia would not, necessarily, be expected to confound comparability of risk factor relationships between samples. Next, in a menopause-stratified analysis of the NHES-Thailand dataset, increasing in FPG was independently associated with decreasing HDL cholesterol specifically in premenopausal women with NGR or with prediabetes but there was no menopause information for HS-England (result not shown). Lastly, one limitation of the study might be the cross-sectional nature of the study. Ascertainment of causation might be difficult that glycemia is the cause or the effect of HDL level.

In summary, our key finding was that variation in glycemia was independently associated with decreasing HDL cholesterol specifically in women and with increasing non-HDL cholesterol in both genders in two markedly different population samples. That increasing glycaemia is associated with an adverse, significant decline in HDL cholesterol, specifically in women may therefore be an association independent of population, and this observation is consistent with reports that women lose their relative protection from CVD on developing impaired glucose homeostasis. The mechanisms of gender differences in relationships between glycemia and lipid profiles should be explored in further studies.

## Methods

### Study populations

The present study used data from two cross-sectional national population samples: the NHES-Thailand, 2014 and the HS-England, 2014. The NHES-Thailand 2014 employed a four-stage sampling process as described previously^34^. The HS-England 2014 surveyed a representative samples of those in private dwellings^35^, with a two-stage stratified random sampling method used, as described previously^36^. The NHES-Thailand 2014 was approved by the Ethical Review Committee for Research in Human Subjects, Faculty of Medicine, Ramathibodi Hospital, Mahidol University (MURA2013/323 S_1–2_NOV_18_) and ethical approval for HS-England 2014 was obtained from the Oxford A Research Ethics Committee (12/SC/0317). The procedures were in accordance with the standards of the ethics committee of both institutes with the Declaration of Helsinki 1975. Written informed consent was obtained from all study participants in the both NHES-Thailand and HS-England 2014 national survey. However, data were analysed anonymously and no consent for this analysis was sought.

### Procedures and measurements

Both NHES-Thailand and HS-England recorded extensive information on participants. Variables relevant to the present analysis included, weight and height, which were measured using standard procedures. Body mass index (BMI) was calculated as weight/height^2^ (kg/m^2^). Waist circumference was measured between the lower rib margin and the iliac crest with end-of-expiration gentle tightening of the tape measure. The mean of two closest measurements of three was used in the analysis as described previously^35–37^. Both surveys recorded participants' current medications and for the present analysis antihypertensive and lipid-lowering medication use information was extracted.

For NHES-Thailand 2014, blood samples were obtained from participants who were asked to fast overnight for 8 h for measurement of FPG, total- and HDL cholesterol. For HS-England 2014, blood samples were taken in the non-fasted state for measurement of HbA1c, total cholesterol and HDL cholesterol. FPG was measured by an enzymatic hexokinase method and HbA1c by IFCC-approved methodology and standardization, using a high performance liquid chromatography system with Tosoh G8 analyser, Tosoh Bioscience, Inc. Serum total- and HDL cholesterol were measured by standard routine laboratory procedures in both countries. Non-HDL cholesterol was calculated as total cholesterol minus HDL cholesterol***.*** Low HDL cholesterol was classified by < 1.0 mmol/L in women and < 1.2 mmol/L in men.

Glucose homeostasis status was categorized according to current national criteria^38,39^ as follows: normal glucose homeostasis (NGH), FPG < 5.6 mmol/L or HbA1c < 5.7% (39 mmol/mol), mild impaired glucose homeostasis (IGH)—IGH-mild (prediabetes stage 2a), FPG 5.6 to < 6.1 mmol/L or HbA1c 5.7 to < 6.0% (39 to < 42 mmol/mol), severe IGH (prediabetes stage 2b): FPG 6.1 to < 7.0 mmol/L or HbA1c 6.0 to < 6.5% (42 to < 48 mmol/mol), and diabetes, FPG ≥ 7.0 mmol/L or HbA1c ≥ 6.5% (48 mmol/mol) and/or currently taking a glucose lowering agent and/or self-reported previous diabetes diagnosis by doctor, Table 1.

### Lifestyle factors

Participants were distinguished as smokers by self-reported cigarette smoking and as drinkers by self-reported drinking of alcohol in the previous 12 months. Regarding physical status, participants were distinguished as undertaking moderate to vigorous physical activity, in the NHES-Thailand, using the Global Physical Activity Questionnaire version 2 (World Health Organization) with physically active defined as having at least moderate to high level of physical activity as described previously^40,41^. Similarly, in HS-England, physical activity was assessed using the Short International Physical activity questionnaire. People engaged in moderate to vigorous intensity physical activity (MVPA, intensity ≥ 3.0 METs) at least 150 min/week were considered to be physically active^42,43^.

### Statistical analysis

The analysis took into account the complex survey design, with both datasets weighted according to the inverse of probability of being sampled based on the 2014 registered Thai or UK English populations thus reducing non-response bias through weighting. In each population dataset separately, individual characteristics and measurements were compared between the categories of glucose homeostasis status: NGH, IGH-mild IGH-severe, and diabetes. Continuous, normally-distributed variables (FPG, HbA1c, age, BMI, waist circumference, HDL-, and non-HDL cholesterol) were summarized as for un-standardised mean ± standard error of mean (SEM), and categorical variables by un-standardised percentage and standard error of the proportion (SEP). Significant variation across glucose homeostasis categories was identified for normally distributed continuous variables by ANOVA and for categorical variables by chi square test (Table 2). Variation across glucose homeostasis categories was evaluated in the NHES-Thailand and HS-England data and in men and women separately. In the gender and dataset separated analysis, the relationships between lipids and glycemia were explored by multivariable linear regression analysis. The independent contributions of variation in glycemia to variation in HDL- and non HDL-cholesterol were then explored in the total population and, separately within the three categories of glycaemia: NGH, IGH (IGH-mild and IGH-severe combined) and diabetes, with adjustment for age, BMI, waist circumference, lipid lowering agent use, current smoking/drinking and active physical status. Subgroup analyses were also carried out in each category of IGH (IGH-mild and IGH-severe) and, for women in the NHES-Thailand data, according to pre- or post-menopausal status. The mean HDL-cholesterol, percent differences and the proportion of low HDL-cholesterol have been standardized by direct age-standardized method using the WHO standard population 2001 (Supplementary Table S1). All statistical analyses were performed using Stata version 13.0 software (Stata Corp, College Station, TX, USA). The significance level was two-sided, and the threshold for statistical significance was set at < 0.05, with no correction for multiple testing as the associations investigated were heavily weighted according to previous evidence^44^.

### Ethic approval and consent to participate

The NHES-Thailand 2014 was approved by the Ethical Review Committee for Research in Human Subjects, Faculty of Medicine, Ramathibodi Hospital, Mahidol University (MURA2013/323 S1-2NOV18) and ethical approval for HS-England 2014 was obtained from the Oxford A Research Ethics Committee (12/SC/0317).

## Supplementary Information

Supplementary Information.

## Data Availability

For HSE 2014, *Health Survey for England, 2014*. [data collection] are available on UK Data Service. SN: 7919, http://doi.org/10.5255/UKDA-SN-7919-3.

## Abbreviations

NGH: Normal glucose homeostasis
IGH: Impaired glucose homeostasis
NHES-Thailand: The National Health Examination Survey for Thailand
HS-England: The Health Survey for England
CVD: Cardiovascular disease
UK: United Kingdom
NHES IV: National Health Examination Survey IV
DECODA: Diabetes Epidemiology: Collaborative analysis of Diagnostic criteria in Asia
DECODE: Diabetes Epidemiology: Collaborative analysis of Diagnostic criteria in Europe
FPG: Fasting plasma glucose
OGTT-2 h glucose: Oral glucose tolerance test 2-h plasma glucose
MVPA: Moderate to vigorous intensity physical activity
