# Supplementary material for: Increasing glycaemia is associated with a significant decline in HDL cholesterol in women with prediabetes in two national populations
Source: Sci Rep. 2021 Jun 9;11:12194. doi: 10.1038/s41598-021-91075-9 (PMC8190299; doi:10.1038/s41598-021-91075-9)
Supplement: Supplementary file 1 — Supplementary Information. [file 41598_2021_91075_MOESM1_ESM.docx]

SUPPLEMENTARY INFORMATION

**Increasing glycaemia is associated with a significant decline in HDL cholesterol in women with prediabetes in two national populations.**

Chaiwat Washirasaksiri^1^, Weerachai Srivanichakorn^1,2^, Ian F Godsland^2^, Chayanis Kositamongkol^1^, Suwat Chariyalertsak^3^, Pattapong Kessomboon^4^, Sawitri Assanangkornchai^5^, Surasak Taneepanichskul^6^, Nareemarn Neelapaichit^7^, Pochamana Phisalprapa^1^, Desmond G Johnston^2^, Nick S Oliver^2^, Wichai Aekplakorn^8^

^1^Department of Medicine, Faculty of Medicine Siriraj Hospital, Mahidol University, Bangkok, Thailand.

^2^ Department of Metabolism, Digestion and Reproduction, Faculty of Medicine, Imperial College, London SW7 2AZ, UK

^3^Faculty of Public Health, Chiang Mai University, Chiang Mai, Thailand

^4^Faculty of Medicine, Khon Kaen University, Khon Kaen, Thailand

^5^Epidemiology Unit, Faculty of Medicine, Prince of Songkla University, Songkhla, Thailand

^6^College of Public Health Sciences, Chulalongkorn University, Bangkok, Thailand

^7^Ramathibodi School of Nursing, Faculty of Medicine, Ramathibodi Hospital, Mahidol University, Bangkok, Thailand

^8^Department of Community Medicine, Faculty of Medicine Ramathibodi Hospital, Mahidol University, Rama VI Rd., Ratchathewi, Bangkok, Thailand

**Correspondence to:** Correspondence should be addressed to **Weerachai Srivanichakorn**

ORCID identifiers; https://orcid.org/0000-0001-9581-7117

Email: [weerachai.srv@mahidol.ac.th](mailto:weerachai.srv@mahidol.ac.th), [w.srivanichakorn@imperial.ac.uk](mailto:w.srivanichakorn@imperial.ac.uk),

Telephone**:** +66 (0) 24197190, Department of Medicine, Faculty of Medicine Siriraj Hospital, Mahidol University, 2 Wang Lang Road, Bangkok Noi, Bangkok, 10700, Thailand.

**Supplementary Table**

**Supplementary Table S1: Percent difference in HDL-c and percentage of abnormally low HDL- c with direct age-standardized method using WHO standard population**

| **Parameters** |  | **NGH** | **IGH - mild** | **IGH - severe** | **Diabetes** | **p^*^** |
| --- | --- | --- | --- | --- | --- | --- |
| **Δ HDL-c,** mmol/L |  |  |  |  |  |  |
| NHES-THAILAND | M | 1.24± 0.01 | -7.9% | -5.2% | -10.6% | <0.001 |
|  | F | 1.36 ± 0.01 | -4.7% | -8.8% | -5.6% | <0.001 |
| HS-ENGLAND | M | 1.43 ± 0.01 | -4.7% | -10.2% | -19.9% | <0.001 |
|  | F | 1.68 ± 0.01 | -8.2% | -14.6% | -13.6% | <0.001 |
| **% low HDL-c**  (M<1.0, F<1.2 mmol/L) |  |  |  |  |  |  |
| NHES-THAILAND | M | 25 (0.01) | 35 (0.03) | 31 (0.06) | 39 (0.06) | <0.001 |
|  | F | 36 (0.01) | 41 (0.04) | 44 (0.06) | 44 (0.05) | <0.001 |
| HS-ENGLAND | M | 9 (0.01) | 23 (0.09) | 8 (0.02) | 13 (0.04) | <0.001 |
|  | F | 9 (0.01) | 12 (0.02) | 23 (0.10) | 32 (0.06) | <0.001 |

M; male, F; Female NGH; normal glucose homeostasis (FPG <5.6 mmol/L (Thai) or HbA1c < 5.7% (UK)), IGH-mild; mild impaired glucose homeostasis (FPG 5.6-<6.1 mmol/L (Thai) or HbA1c 39-<42 mmol/mol (UK)), IGH-severe: severe impaired glucose homeostasis (FPG 6.1-<7.0 mmol/L (Thai) or HbA1c 42-<48 mmol/mol (UK)), diabetes, FPG ≥7.0 mmol/L or HbA1c ≥ 48 mmol/mol and/or currently taking a glucose lowering agent and/or self-reported previous diabetes diagnosis by doctor, the National Health Examination Survey for Thailand,

**Supplementary Table S2: Non-lipid lowering agent use group: regression coefficients (95% CI) of FPG and covariates associated with HDL-c and Non HDL-c by glycemic categories and gender in the Thai National Health Examination Survey**

| **HEST 2014** | **Male** | | | | **Female** | | | | |
| --- | --- | --- | --- | --- | --- | --- | --- | --- | --- |
| ***HDL-c*** | **Overall** | **NGH** | **IGH** | **Diabetes** | **overall** | **NGH** | **IGH** | **Diabetes** |  |
| **FPG** | -0.004  (-0.015,0.006)^0.4^ | 0.004  (-0.015,0.023)^0.6^ | 0.075  (-0.004,0.154)^0.06^ | 0.013  (-0.002,0.027)^0.09^ | -0.010  (-0.017, -0.002)^0.01^ | -0.027  (-.0480,-0.007)^0.01^ | -0.084  (-0.168,-0.0005)^0.04^ | -0.001  (-0.010,0.009)^0.8^ |  |
| **age** | -0.001  (-0.002,0.0001)^0.06^ | -0.001  (-0.002,0.0001)^0.08^ | 0.001  (-0.001,0.003)^0.2^ | 0.001  (-0.003,0.005)^0.5^ | 0.0001  (-0.001,0.001)^0.8^ | -0.0002  (-0.001,0.001)^0.7^ | 0.002  (-0.0001,0.005)^0.05^ | 0.0001  (-0.003,0.004)^0.9^ |  |
| **Waist circumference** | -0.009  (-0.010,-0.007)^<0.001^ | -0.010  (-0.010,-0.008)^<0.001^ | -0.008  (-0.011,-0.006)^<0.001^ | -0.003  (-0.007,0.002)^0.2^ | -0.008  (-0.009,-0.007)^<0.001^ | -0.008  (-0.009,-0.007)^<0.001^ | -0.005  (-0.008,-0.003)^<0.001^ | -0.007  (-0.011,-0.002)^0.002^ |  |
| ***Non HDL-c*** | **Overall** | **NGR** | **IGH** | **Diabetes** | **Overall** | **NGR** | **IGH** | **Diabetes** |  |
| **FPG** | 0.031  (0.0004,0.061)^0.04^ | 0.062  (-0.022,0.145)^0.1^ | 0.108  (-0.158,0.370)^0.4^ | 0.070  (0.017, 0.122)^0.009^ | 0.059  (0.034,0.084)^<0.001^ | 0.157  (0.094,0.219)^<0.001^ | -0.033  (-0.261,0.196)^0.7^ | 0.096  (0.062,0.130)^<0.001^ |  |
| **age** | 0.008  (0.005,0.011)^<0.001^ | 0.010  (0.007,0.013)^<0.001^ | 0.005  (-0.003,0.012)^0.2^ | -0.010  (-0.024,0.004)^0.1^ | 0.015  (0.012,0.017)^<0.001^ | 0.015  (0.012,0.018)^<0.001^ | 0.015  (0.007,0.022)^<0.001^ | 0.010  (-0.001,0.021)^0.08^ |  |
| **Waist circumference** | 0.025  (0.021,0.029)^<0.001^ | 0.024  (0.019,0.029)^<0.001^ | 0.029  (0.020,0.038)^<0.001^ | 0.030  (0.010,0.040)^<0.001^ | 0.015  (0.012,0.018)^<0.001^ | 0.015  (0.011,0.019)^<0.001^ | 0.012  (0.004,0.020)^0.002^ | 0.020  (0.008,0.032)^0.001^ |  |

FPG; fasting plasma glucose, HDL-c; high density lipoprotein cholesterol, NGH; normal glucose homeostasis (FPG <5.6 mmol/L), IGH; impaired glucose homeostasis (FPG 5.6-<7.0 mmol/L),diabetes, FPG ≥7.0 mmol/L and/or currently taking a glucose lowering agent and/or self-reported previous diabetes diagnosis by doctor. Predictor variables included for in each multivariable model included age, waist circumference, smoking, alcohol status, total energy intake and physical activity status**.**

**Supplementary Table S3: Lipid lowering agent use group: regression coefficients (95% CI) of FPG and covariates associated with HDL-c and Non HDL-c by glycemic categories and gender in the Thai National Health Examination Survey**

| **HEST 2014** | **Male** | | | | **Female** | | | |
| --- | --- | --- | --- | --- | --- | --- | --- | --- |
| ***HDL-c*** | **Overall** | **NGH** | **IGH** | **Diabetes** | **overall** | **NGH** | **IGH** | **Diabetes** |
| **FPG** | -0.011  (-.026,0.003)^0.1^ | -0.125  (-0.233,-.0162)^0.024^ | -0.059  (-0.292,0.174)^0.6^ | 0.007  (-0.005,0.019)^0.2^ | -0.015  (-0.027,-0.003)^0.01^ | 0.030  (-0.072,0.132)^0.5^ | -0.010  (-0.135,0.111)^0.8^ | -0.006  (-0.020, 0.008)^0.4^ |
| **age** | -0.0007  (-0.005,0.004)^0.7^ | -0.001  (-0.006,0.004)^0.7^ | 0.005  (-0.001,0.011)^0.1^ | -0.0002  (-0.005,0.005)^0.9^ | -0.001  (-0.004,0.002)^0.5^ | 0.0003  (-0.004,0.004)^0.8^ | -0.0003  (-0.005,0.005)^0.9^ | -0.002  (-0.007,0.002)^0.3^ |
| **Waist circumference** | -0.003  (-0.007,0.002)^0.2^ | 0.005  (-0.001,0.011)^0.1^ | -0.008  (-0.014,-0.001)^0.01^ | -0.007  (-0.011,-0.002)^0.004^ | -0.007  (-0.009,0.004)^<0.001^ | -0.008  (-0.011, 0.005)^<0.001^ | -0.007  (-0.012, -0.001)^0.01^ | -0.005  (-0.009,-0.0003)^0.03^ |
| ***Non HDL-c*** | **Overall** | **NGR** | **IGH** | **Diabetes** | **Overall** | **NGR** | **IGH** | **Diabetes** |
| **FPG** | 0.058  (-0.050,0.170)^0.2^ | -0.242  (-0.766,0.283)^0.3^ | -0.265  (-0.806,0.276)^0.3^ | 0.124  (0.029,0.219)^0.01^ | 0.015  (-0.031,0.061)^0.5^ | 0.219  (-0.044,0.482)^0.1^ | -0.472  (-0.992,0.049)^0.07^ | 0.068  (0.009,0.128)^0.02^ |
| **age** | -0.016  (-0.030,-0.003)^0.01^ | -0.008  (-0.027,0.011)^0.4^ | -0.032  (-0.061,-0.003)^0.03^ | -0.010  (-0.037,0.011)^0.2^ | 0.001  (-0.007,0.009)^0.8^ | -0.004  (-0.015,0.006)^0.4^ | 0.002  (-0.019,0.023)^0.8^ | 0.011  (-0.004,0.027)^0.1^ |
| **Waist circumference** | 0.024  (-0.001,0.050)^0.06^ | 0.021  (0.002,0.040)^0.03^ | 0.049  ( -0.003, 0.101)^0.06^ | -0.001  (-0.020,0.017)^0.8^ | 0.001  (-0.007,0.009)^0.8^ | -0.002  (-0.013,0.008)^0.6^ | 0.003  (-0.015,0.021)^0.76^ | 0.009  (-0.010,0.020)^0.2^ |

FPG; fasting plasma glucose, HDL-c; high density lipoprotein cholesterol NGH; normal glucose homeostasis (FPG <5.6 mmol/L), IGH; impaired glucose homeostasis (FPG 5.6-<7.0 mmol/L), diabetes, FPG ≥7.0 mmol/L and/or currently taking a glucose lowering agent and/or self-reported previous diabetes diagnosis by doctor. Predictor variables included for in each multivariable model included age, waist circumference, smoking, alcohol status, total energy intake and physical activity status

**Supplementary Table S4: Non-lipid lowering agent use group: regression coefficients (95% CI) of HbA1c and covariates associated with HDL-c and Non HDL-c by glycemic categories and gender in the Health Survey for England**

| **HSE 2014** | **Male** | | | | **Female** | | | |
| --- | --- | --- | --- | --- | --- | --- | --- | --- |
| ***HDL-c*** | **Overall** | **NGH** | **IGH** | **Diabetes** | **overall** | **NGH** | **IGH** | **Diabetes** |
| **HbA1c** | -0.004  (-0.006,-0.001)^0.007^ | 0.006  (-0.004,0.015)^0.2^ | -0.003  (-0.029,0.023)^0.8^ | -0.004  (-0.007,-0.001)^0.01^ | -0.003  (-0.007,0.0003)^0.07^ | -0.006  (-0.015,0.003)^0.1^ | -0.018  (-0.037,0.001)^0.05^ | 0.004  (-0.004,0.011)^0.3^ |
| **age** | 0.004  (0.003,0.006)^<0.001^ | 0.004  (0.003,0.006)^<0.001^ | 0.003  (-0.002,0.008)^0.2^ | -0.001  (-0.007,0.005)^0.7^ | 0.009  (0.008,0.011)^<0.001^ | 0.011  (0.009,0.012)^<0.001^ | 0.008  (0.005,0.012)^<0.001^ | 0.001  (-0.011,0.012)^0.8^ |
| **Waist circumference** | -0.010  (-0.013,-0.008)^<0.001^ | -0.011  (-0.014,-0.008)^<0.001^ | -0.010  (-0.013,-0.006)^<0.001^ | 0.002  (-0.004,0.009)^0.4^ | -0.015  (-0.016, -0.013)^<0.001^ | -0.015  (-0.017, -0.013)^<0.001^ | -0.013  (-0.016, -0.010)^<0.001^ | -0.010  (-0.024,0.004)^0.1^ |
| ***Non HDL-c*** | **Overall** | **NGR** | **IGH** | **Diabetes** | **Overall** | **NGR** | **IGH** | **Diabetes** |
| **HbA1c** | 0.012  (-0.003,0.026)^0.1^ | 0.029  (0.004,0.053)^0.02^ | 0.031  (-0.038,0.100)^0.3^ | 0.021  (-0.008,0.051)^0.1^ | 0.014  (0.003, 0.025)^0.01^ | 0.051  (0.031,0.072)^<0.001^ | -0.004  (-0.058,0.051)^0.8^ | -0.015  (-0.038,0.009)^0.2^ |
| **age** | 0.011  (0.006,0.015)^<0.001^ | 0.012  (0.007,0.017)^<0.001^ | 0.0005  (-0.011,0.012)^0.9^ | -0.020  (-0.045,0.005)^0.1^ | 0.028  (0.024,0.031)^<0.001^ | 0.024  (0.020,0.028)^<0.001^ | 0.022  (0.014,0.031)^<0.001^ | 0.039  (0.002,0.077)^0.04^ |
| **Waist circumference** | 0.035  (0.030, 0.041)^<0.001^ | 0.038  (0.031,0.044)^<0.001^ | 0.021  (0.008,0.033)^0.001^ | 0.044  (0.016,0.073)^0.003^ | 0.019  (0.015,0.022)^<0.001^ | 0.018  (0.014,0.023)^<0.001^ | 0.017  (0.009,0.024)^<0.001^ | -0.005  (-0.030,0.020)^0.6^ |

HbA1c; Hemoglobin A1c, HDL-c; high density lipoprotein cholesterol, NGH; normal glucose homeostasis (HbA1c < 5.7%), IGH; impaired glucose homeostasis **(**HbA1c 39-<42 mmol/mol), diabetes, HbA1c ≥ 48 mmol/mol and/or currently taking a glucose lowering agent and/or self-reported previous diabetes diagnosis by doctor. Predictor variables included for in each multivariable model included age, waist circumference, smoking, alcohol status, total energy intake and physical activity status

**Supplementary Table S5: Lipid lowering agent use group: regression coefficients (95% CI) of HbA1c and covariates associated with HDL-c and Non HDL-c by glycemic categories and gender in the Health Survey for England**

| **HSE 2014** | **Male** | | | | **Female** | | | |
| --- | --- | --- | --- | --- | --- | --- | --- | --- |
| ***HDL-c*** | **Overall** | **NGH** | **IGH** | **Diabetes** | **overall** | **NGH** | **IGH** | **Diabetes** |
| **HbA1c** | -0.003  (-0.007,0.002)^0.2^ | -0.010  (-0.038,0.018)^0.4^ | 0.007  (-0.027,0.042)^0.6^ | 0.001  (-0.004,0.007)^0.6^ | -0.004  (-0.008,-0.001)^0.01^ | 0.006  (-0.03,0.043)^0.7^ | -0.033  (-0.087,0.021)^0.2^ | -0.002  (-0.006,0.003)^0.5^ |
| **age** | 0.004  (-0.003,0.011)^0.2^ | 0.015  (0.004,0.025)^0.005^ | -0.001  (-0.012,0.011)^0.8^ | -0.002  (-0.011,0.007)^0.6^ | 0.005  (-0.002,0.013)^0.1^ | 0.012  (0.0003,0.0237)^0.04^ | 0.003  (-0.009,0.015)^0.6^ | 0.0003  (-0.012,0.013)^0.9^ |
| **Waist circumference** | -0.009  (-0.014,-0.005)^<0.001^ | -0.006  (-0.0142,0.002)^0.1^ | -0.009  (-0.016,-0.003)^0.006^ | -0.008  (-0.014,-0.003)^0.006^ | -0.010  (-0.015,-0.004)^0.001^ | -0.008  (-0.0154,-0.0002)^0.04^ | -0.0153556  (-0.028,-0.003)^0.01^ | -0.003  (-0.015,0.009)^0.6^ |
| ***Non HDL-c*** | **Overall** | **NGR** | **IGH** | **Diabetes** | **Overall** | **NGR** | **IGH** | **Diabetes** |
| **HbA1c** | -0.012  (-0.023,-0.001)^0.02^ | 0.118  (0.037,0.198)^0.005^ | -0.033  (-0.132,0.066)^0.5^ | 0.009  (-0.005,0.023)^0.1^ | -0.001  (-0.010,0.010)^0.8^ | 0.036  (-0.059,0.132)^0.4^ | -0.084  (-0.159,-0.008)^0.03^ | 0.002  (-0.012,0.016)^0.7^ |
| **age** | -0.034  (-0.051,-0.017^)<0.001^ | -0.046  (-0.07,-0.024)^<0.001^ | -0.059  (-0.09,-0.028)^<0.001^ | -0.006  (-0.034,0.023)^0.6^ | -0.011  (-0.03,0.010)^0.2^ | -0.009  (-0.033,0.015)^0.4^ | 0.005  (-0.022,0.032)^0.7^ | -0.027  (-0.07,0.02)^0.2^ |
| **Waist circumference** | 0.008  (-0.003,0.020)^0.1^ | -0.007  (-0.023,0.009)^0.3^ | 0.022  (-0.002,0.047)^0.07^ | 0.008  (-0.007,0.023)^0.2^ | 0.006  (-0.007,0.020)^0.3^ | -0.004  (-0.03,0.02)^0.7^ | 0.0214  (0.001,0.042)^0.03^ | 0.013  (-0.012,0.039)^0.3^ |

HbA1c; Hemoglobin A1c, HDL-c; high density lipoprotein cholesterol, NGH; normal glucose homeostasis (HbA1c < 5.7%), IGH; impaired glucose homeostasis **(**HbA1c 39-<42 mmol/mol), diabetes; HbA1c ≥ 48 mmol/mol and/or currently taking a glucose lowering agent and/or self-reported previous diabetes diagnosis by doctor. Predictor variables included for in each multivariable model included age, waist circumference, smoking, alcohol status, total energy intake and physical activity status
